# Antimicrobial Resistance and Virulence of *Acinetobacter baumannii*; A Whole-Genome Sequencing Perspective from a Croatian Intensive Care Unit

**DOI:** 10.3390/antibiotics15080814

**Published:** 2026-08-20

**Authors:** Marija Cavka, Marija Kvesic Ivankovic, Ana Maravic, Mia Dzelalija, Jelena Marinovic, Ivana Goic-Barisic, Marija Tonkic, Toni Kljakovic Gaspic, Anita Novak

**Affiliations:** 1 Department of Anesthesiology and Intensive Care, University Hospital of Split, Spinciceva 1, 21 000 Split, Croatia; mcavka@kbsplit.hr (M.C.); tkgaspic@kbsplit.hr (T.K.G.); 2 Institute of Oceanography and Fisheries, Setaliste Ivana Mestrovica 63, 21 000 Split, Croatia; 3 Department of Biology, Faculty of Science, University of Split, Rudera Boskovica 33, 21 000 Split, Croatia; mdzelalij@pmfst.hr; 4 Department of Clinical Microbiology, University Hospital of Split, Spinciceva 1, 21 000 Split, Croatia; jelena.marinovic@kbsplit.hr (J.M.); igoic@kbsplit.hr (I.G.-B.); mtonkic@kbsplit.hr (M.T.); 5 School of Medicine, University of Split, Soltanska 2A, 21 000 Split, Croatia

**Keywords:** *Acinetobacter baumannii*, Intesive Care Unit, antimicrobial resistance, whole-genome sequencing

## Abstract

Background/Objectives: *Acinetobacter baumannii* is a major cause of infections in Intensive Care Units (ICUs), driving mortality through high-level antimicrobial resistance. This study utilized whole-genome sequencing (WGS) to evaluate the phenotypic, genotypic and virulence features of carbapenem-resistant *A. baumannii* (CRAB), which has caused ventilator-associated pneumonia/tracheobronchitis (VAP/VAT) in the ICU of the University Hospital of Split, Croatia. Methods: Over 1 year, lower respiratory tract specimens from 79 VAP/VAT patients were analyzed. CRAB isolates were identified via MALDI-TOF MS and evaluated for antimicrobial susceptibility, and a representative subset underwent WGS and multilocus sequence typing (MLST). Results: Out of 106 specimens, 18 non-duplicate CRAB strains were isolated. Five isolates underwent genomic analysis, identifying two globally distributed, high-risk Pasteur lineages: ST2 and ST492. These lineages displayed distinct resistomes: ST2 carried *bla*_OXA-23_ and *bla*_ADC-73_, while ST492 harbored plasmid-borne *bla*_OXA-72_ (Rep3-T1/AB082 cluster) and *bla*_ADC-30_. All isolates shared aminoglycoside/macrolide-resistance genes, conserved efflux pumps, and virulence determinants (*bau*, *bas*, *ent*, *bar*) crucial for acinetobactin synthesis and respiratory colonization. Phylogenetic analysis confirmed regional circulation and genetic links to neighboring countries. Conclusions: This study highlights the evolutionary dynamics of endemic CRAB lineages in a Croatian ICU and their global dissemination, which poses a critical threat, demanding strict infection control and novel therapeutics.

## 1. Introduction

*Acinetobacter baumannii* (*A. baumannii*) has emerged as one of the most challenging opportunistic pathogens globally and is frequently associated with a wide spectrum of severe healthcare-associated infections (HAIs), including ventilator-associated pneumonia (VAP), sepsis, and skin and soft tissue infections. Due to its remarkable ability to survive on abiotic surfaces and its propensity for acquiring diverse resistance mechanisms, the World Health Organization (WHO) has classified carbapenem-resistant *A. baumannii* (CRAB) as a “critical priority” pathogen for which new therapeutic strategies are urgently required [1,2,3]. The clinical management of *A. baumannii* is increasingly compromised by the prevalence of multidrug-resistant (MDR) and extensively drug-resistant (XDR) strains. Infections caused by these phenotypes are associated with limited therapeutic options and high mortality rates from bacteremia and VAP, reaching up to 50% in the Intensive Care Unit (ICU) setting [4,5].

While healthcare-associated exposure remains the primary catalyst for *A. baumannii* transmission, patient susceptibility is driven by a multifactorial intersection of host biology and medical procedures [6,7]. Specifically, advanced age, severe underlying pathologies, and compromised immunity significantly elevate infection risk. Furthermore, invasive interventions serve as a portal of entry for colonization and subsequent invasive disease. Prolonged ICU stays, recent history of dialysis, and prior exposure to broad-spectrum antimicrobials (specifically third-generation cephalosporins, fluoroquinolones, and carbapenems) within the preceding 90 days have been identified as critical drivers for the acquisition of MDR strains [6].

The clinical persistence of this pathogen is largely attributed to its extraordinary genetic plasticity, which facilitates the rapid development of antimicrobial resistance. While intrinsic resistance mechanisms provide a baseline defense, *A. baumannii* can rapidly elevate its minimum inhibitory concentrations (MICs) to critical antibiotics, like carbapenems [6]. This is achieved either through spontaneous chromosomal mutations or via the horizontal acquisition of exogenous-resistance genes through mobile genetic elements [6,8]. Beyond its extensive antibiotic resistance profiles, *A. baumannii* possesses a robust repertoire of virulence factors [1]. The pathogenicity of *A. baumannii* is mediated by a sophisticated array of cell-surface and secreted components. Among these, outer membrane porins (Omps) play a critical role in maintaining outer membrane integrity and regulating cellular permeability. The predominant porin, OmpA, is a multifunctional virulence factor that facilitates adhesion to host epithelial cells, promotes biofilm formation on biotic and abiotic surfaces, and contributes to complement resistance. Additionally, other crucial structures, such as the capsular polysaccharide, specialized protein secretion systems, and the immunoreactive lipopolysaccharide (LPS), collectively shield the pathogen from host immune responses and mediate tissue invasion [9].

Clinically, pneumonia represents the most prevalent nosocomial infection caused by *A. baumannii*, particularly in ICU patients undergoing mechanical ventilation, where it serves as a leading cause of VAP and ventilator-associated tracheobronchitis (VAT) [10]. Characterizing the resistome, pathogenicity, and clinical risk factors is fundamental to ensuring timely targeted therapy and implementing robust infection control and antimicrobial stewardship frameworks. In recent years, advances in high-throughput sequencing technologies have revolutionized our understanding of these mechanisms. The expanding availability of whole-genome sequencing (WGS) data, combined with cutting-edge bioinformatics tools, has established comparative genomics as a definitive approach to dissecting the complex resistome and virulome of this pathogen [11].

To better understand the global epidemiology and genetic features of these high-risk strains, genomic surveillance is essential. In this study, we present the phenotypic and genotypic characterization of *A. baumannii* isolates recovered from patients diagnosed with VAP/VAT in the ICU at the University Hospital of Split, Croatia, which has been recognized as an endemic setting for carbapenem-resistant *Acinetobacter baumannii* (CRAB) for decades. The first isolates were identified in 2009, marking the first documented detection of OXA-72-producing *A. baumannii* in Southeast Europe, while longitudinal surveillance of the pathogen has demonstrated the emergence of both OXA-72- and OXA-23-resistance determinants, accompanied by an evolutionary progression toward an XDR profile [5,12]. Utilizing WGS, the aim of this study was to characterize representative CRAB isolates from a Croatian ICU by integrating complementary genomic analyses to determine their phylogenetic relationships, investigate the genetic basis and genomic context of antimicrobial resistance, and place the identified lineages within a regional and global phylogenomic framework.

## 2. Results

### 2.1. Phenotypic Antimicrobial Susceptibility Testing (AST)

**Figure 1:**
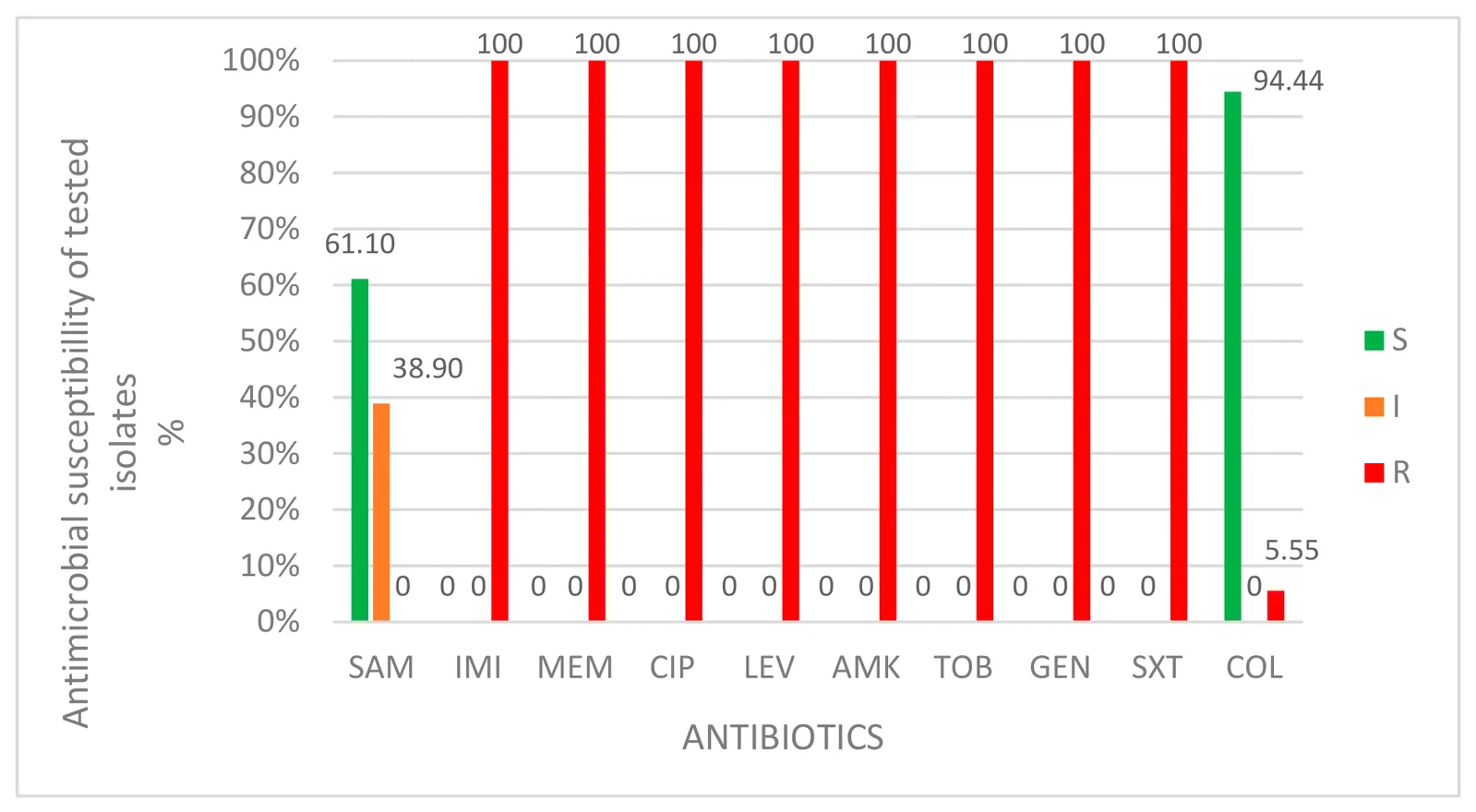
Antimicrobial susceptibility pattern of 18 *A. baumannii* isolates. Note: SAM, ampicillin–sulbactam; IMI, imipenem; MEM, meropenem; CIP, ciprofloxacin; LEV, levofloxacin; AMK, amikacin; TOB, tobramycin; GEN, gentamicin; SXT, trimethoprim–sulfamethoxazole; COL, colistin; S, susceptible; I, susceptible, increased exposure; R, resistant.

The study included 106 lower respiratory tract samples collected from 79 patients who met the diagnostic criteria for VAP and VAT. Among the analyzed samples, 18 non-duplicate *A. baumannii* strains were isolated and were subsequently tested for antimicrobial susceptibility (results are shown in Figure 1). Antimicrobial susceptibility testing revealed a 100% resistance rate across all 18 isolates against four major antibiotic classes. Specifically, all strains were fully resistant to carbapenems (meropenem (MEM) and imipenem (IMI)), quinolones (ciprofloxacin (CIP) and levofloxacin (LEV)) and aminoglycosides (amikacin (AMK), tobramycin (TOB) and gentamicin (GEN)). Furthermore, every isolate demonstrated resistance to trimethoprim–sulfamethoxazole (SXT). Susceptibility testing for ampicillin–sulbactam (SAM) revealed that 11 isolates (61.1%) were susceptible, while the remaining 7 (38.9%) were classified as susceptible with increased exposure. Notably, no resistance was detected among the 18 isolates. Among the tested strains, 17 isolates (94.4%) were susceptible to colistin (COL), with minimum inhibitory concentrations (MICs) ranging from 0.5 to 1 µg/mL. Conversely, a single isolate (5.6%) demonstrated COL resistance, with an MIC exceeding 16 µg/mL.

Based on the AST profile, all isolates met the criteria for XDR bacteria, demonstrating uniform resistance to four critical antimicrobial classes: carbapenems, quinolones, aminoglycosides, and SXT, whereas the single COL-resistant isolate exhibited a PDR phenotype.

### 2.2. Whole-Genome Sequencing (WGS) Profiling and MLST Analysis

#### 2.2.1. Multilocus Sequence Typing (MLST) and Phylogenetic Analysis

Whole-genome sequencing was successfully performed for five CRAB isolates (V6–V10). Draft genome assemblies ranged from 3.94 to 4.00 Mb in size, with GC contents varying between 38.88% and 38.99%. Sequencing depth ranged from 199.9× to 484.8×, while N50 values varied between 137,171 bp and 164,822 bp. The number of predicted coding sequences ranged from 3712 to 3794. Detailed assembly statistics and accession numbers are provided in Table S1.

Multi-locus sequence typing identified two different *A. baumannii* lineages among the five sequenced isolates. According to the Pasteur scheme, isolates V6, V8, and V9 belonged to ST2, whereas V7 and V10 were assigned to ST492 (a single-locus variant of ST2). Using the Oxford scheme, isolates V6, V8, and V9 were classified as ST1816/ST195, while V7 and V10 belonged to ST1806/ST208, reflecting the presence of two *gdhB* alleles in each isolate.

MASH analysis of the five *A. baumannii* isolates against 1012 publicly available complete *A. baumannii* RefSeq genomes identified the closest reference genomes for each isolate (Figure S1A). Hierarchical clustering based on MASH distances revealed two major genomic groups among the study isolates. Isolates V6 and V9 formed a highly similar cluster and showed the highest similarity to genome GCF_056115645.1, with very low MASH distances. Isolate V8 exhibited the highest similarity with low MASH distance to genome GCF_056115605.1. In contrast, isolates V7 and V10 formed a separate cluster and were most closely related to genome GCF_055598425.1. Average nucleotide identity (ANI) analysis performed using fastANI confirmed the clustering pattern observed in the MASH dendrogram (Figure S1B). In particular, isolates V6 and V9 remained closely associated, with V8 clustering nearest to this group, whereas V7 and V10 formed a distinct cluster. All isolates displayed very high nucleotide identity (>99.7% ANI) relative to their closest reference genomes, confirming their close genomic relationship to publicly available *A. baumannii* genomes. The observed clustering pattern was consistent with the MLST classification of the isolates.

**Figure 2:**
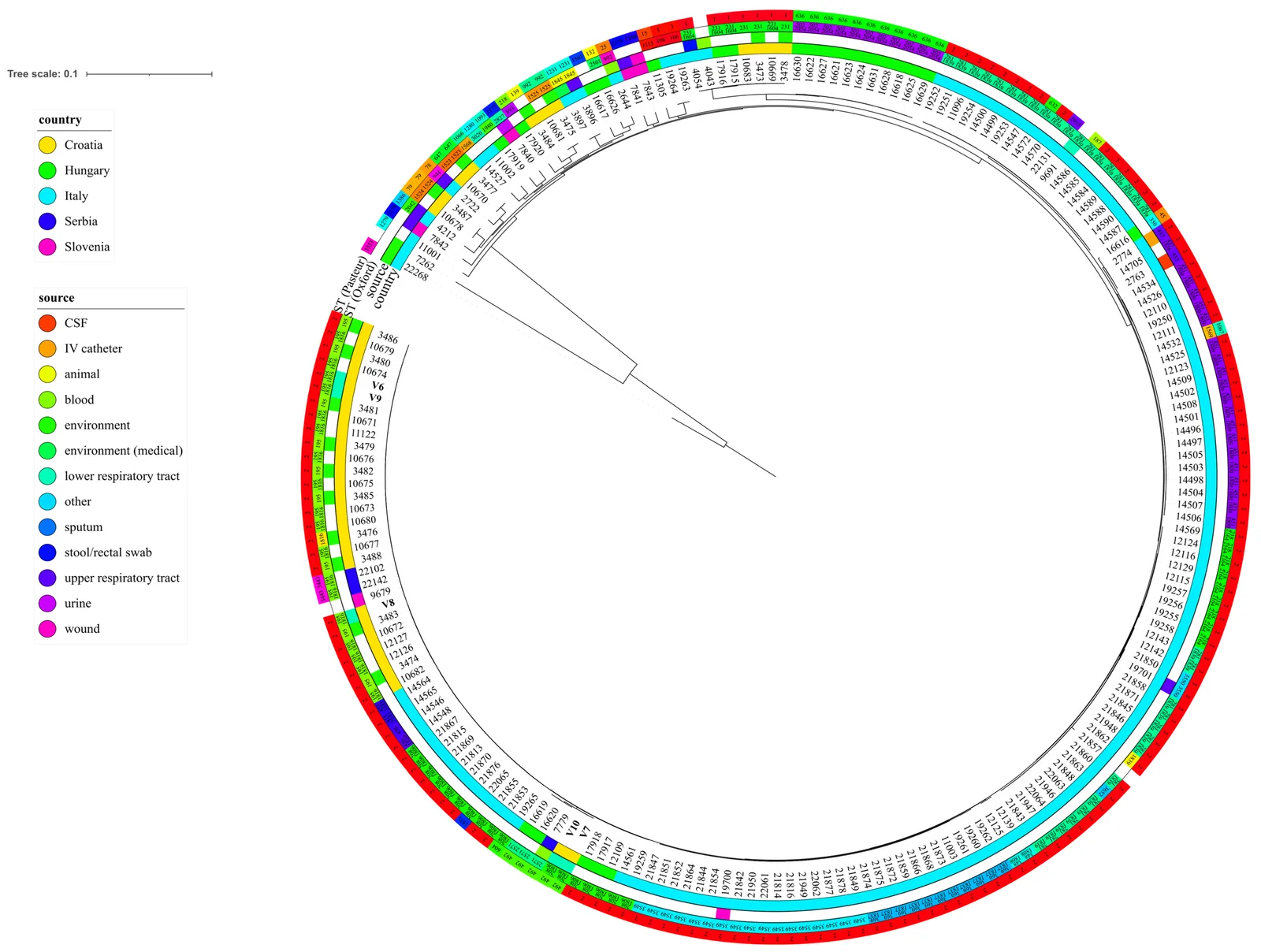
cgMLST-based phylogenetic tree of 299 *A. baumannii* isolates, including five isolates sequenced in this study (V6–V10). Colored rings indicate Pasteur and Oxford MLST sequence type (two outer rings), isolation source (middle ring), and country of origin (inner ring). The genomes generated in this study are highlighted in bold.

A cgMLST-based phylogenetic analysis was performed on 299 *A. baumannii* genomes, including five isolates sequenced in this study and 294 publicly available genomes retrieved from neighboring countries (Figure 2).

Isolates V6, V8, and V9 clustered within the major regional lineage were composed predominantly of Pasteur ST2 isolates, with V6 and V9 showing the closest phylogenetic relationship and V8 forming a neighboring branch within the same lineage. Within the broader regional cluster, isolates V7 and V10 formed a subclade corresponding to Pasteur ST492. This subclade clustered most closely with genomes originating from Serbia and Hungary. Overall, the cgMLST phylogeny was fully concordant with the MLST classification of the study isolates.

Pairwise core-genome SNP analysis provided quantitative support for the phylogenomic relationships among the five isolates (Table S2; Figure S2). Within Pasteur ST2, isolates V6 and V9 differed by only six SNPs, indicating very close genomic relatedness. In contrast, isolate V8 differed from V6 and V9 by 401 and 405 SNPs, respectively, indicating that it belonged to the same ST2 lineage but represented a more divergent genomic variant. The two ST492 isolates, V7 and V10, differed by 15 SNPs and were likewise closely related. Comparisons between ST2 and ST492 isolates yielded substantially greater distances, ranging from 7311 to 7442 SNPs, confirming that these sequence types represent two distinct genomic lineages.

#### 2.2.2. Antimicrobial Resistance Genomic Profile

**Figure 3:**
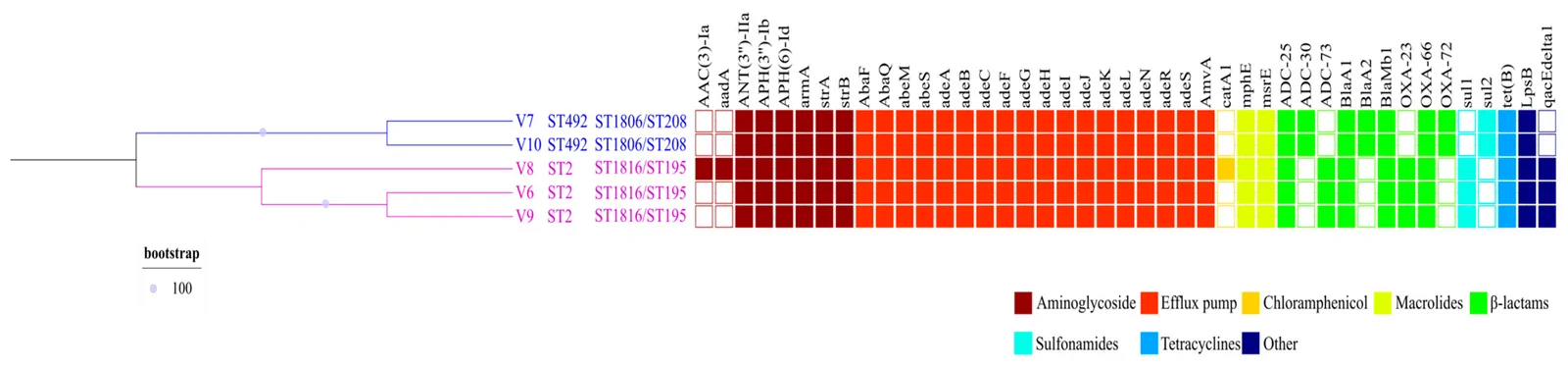
Core genome phylogeny and distribution of antimicrobial resistance genes among the five *A. baumannii* isolates. Sequence types assigned according to the Pasteur and Oxford MLST schemes are indicated next to each isolate. The heatmap shows the presence (colored squares) or absence (white squares) of antimicrobial resistance genes identified by WGS. Antibiotic resistance gene categories are indicated by the color legend.

**Figure 4:**
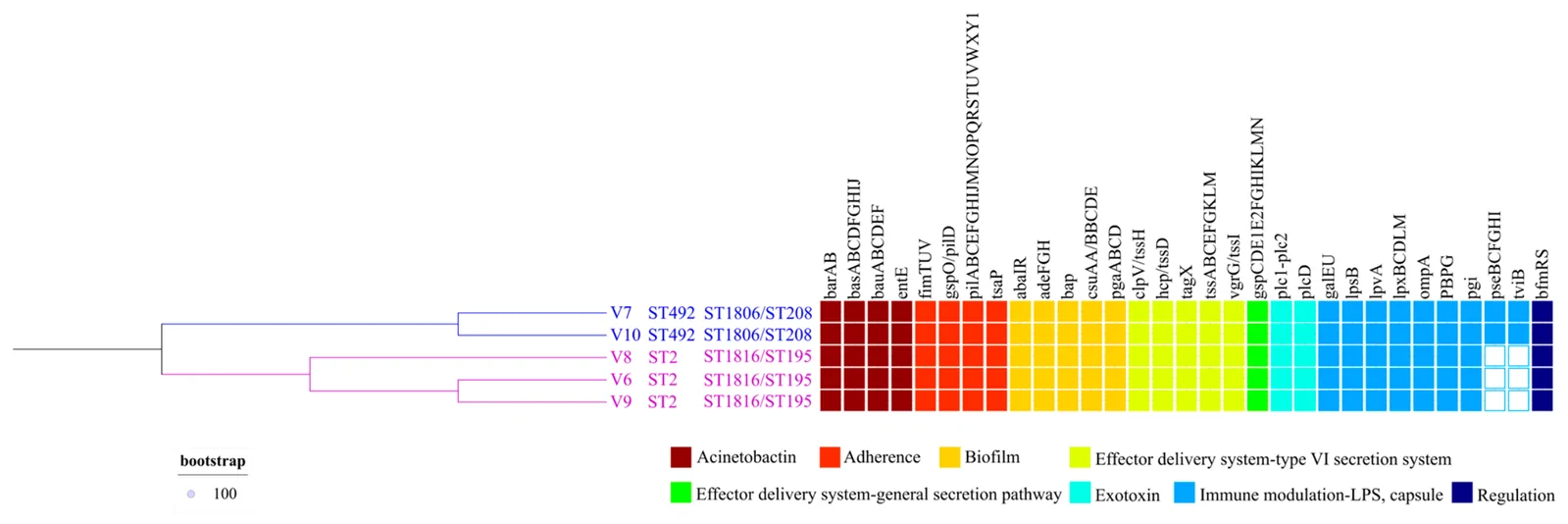
Core genome phylogeny and distribution of virulence-associated genes among the five *A. baumannii* isolates. Sequence types assigned according to the Pasteur and Oxford MLST schemes are indicated next to each isolate. The heatmap shows the presence (colored squares) or absence (white squares) of virulence-associated genes identified using the VFDB database. Functional gene categories are indicated by the color legend.

Panaroo identified a core genome alignment comprising 3,308,470 nucleotide positions, including 8066 parsimony-informative sites, which were used for maximum-likelihood phylogenetic reconstruction (Figure 3 and Figure 4). The resulting phylogeny resolved the five *A. baumannii* isolates into two well-supported clusters corresponding to the ST2 and ST492 lineages. Along with that, genome analysis identified a broad repertoire of acquired and intrinsic antimicrobial resistance determinants in all five isolates (Figure 3), with the overall resistome reflecting their phylogenetic clustering.

All isolates carried the aminoglycoside resistance genes *ant(3″)-IIa*, *aph(3″)-Ib*, *aph(6)-Id*, *armA*, *strA*, and *strB*, together with the macrolide resistance genes *mph(E)* and *msr(E)*. All isolates possessed a conserved repertoire of chromosomally encoded multidrug efflux systems, including the RND-family pumps AdeABC, AdeFGH and AdeIJK, the MATE-family transporter AbeM, the SMR-family transporter AbeS, the MFS-family transporter AmvA, and the ABC transporters AbaF and AbaQ, as well as their associated regulatory genes (AdeL, AdeN, AdeR, and AdeS). The intrinsic β-lactamase *bla*_A1_ was also detected in all genomes, whereas *bla*_A2_ was restricted to the ST492 isolates. Additional resistance determinants, including *aac(3)-Ia*, *aadA,* and *catA1*, were detected only in isolate V8. Phenotypically, this strain differs from the other ST2 strains (V6 and V9) only in its susceptibility to SAM under increased exposure, whereas the latter two were fully susceptible. Furthermore, ST492 strains (V7 and V10) displayed different phenotypic susceptibility profiles to COL. Specifically, V7 was COL-susceptible, while V10 showed resistance to COL.

Different β-lactam resistance profiles were observed between the two MLST groups. The ST2 isolates carried the acquired carbapenemase gene *bla*_OXA-23_ together with *bla*_ADC-73_, whereas the ST492 isolates possessed *bla*_OXA-72_ and *bla*_ADC-30_. The intrinsic *bla*_OXA-66_, a variant of the *bla*_OXA-51-like_ gene, was present in all isolates. Furthermore, *sul1*, together with the disinfectant resistance-associated gene *qacEΔ1*, was detected in the ST2 isolates, whereas *sul2* was identified in the ST492 isolates. Comparative genomic analysis of the phenotypically colistin-resistant isolate V10 against the closely related susceptible isolate V7 and publicly available colistin-resistant and colistin-susceptible genomes did not identify any known genetic determinant associated with the observed colistin-resistant phenotype.

#### 2.2.3. Virulence Genomic Profile

Genome screening against the VFDB database identified a highly conserved virulence gene repertoire among the five *A. baumannii* isolates (Figure 4). Conserved virulence determinants included genes involved in iron acquisition, adherence and type IV pili biogenesis, biofilm formation, type II and type VI secretion systems, phospholipase-associated exotoxins, lipopolysaccharide and capsule biosynthesis, and global virulence regulation.

The overall virulence gene profiles closely mirrored the phylogenetic clustering, with nearly identical repertoires observed within the lineages. The only differences were restricted *pseBCFGHI* cluster and *tviB*, which were detected exclusively in the ST492 isolates.

#### 2.2.4. Genetic Context of Antimicrobial Resistance Determinants

Comparative genomic analyses were performed to investigate the genetic organization and potential mobility of the multi-drug resistance region carrying *armA–msr(E)–mph(E)* and the carbapenemase genes *bla*_OXA-23_ and *bla*_OXA-72_.

**Figure 5:**
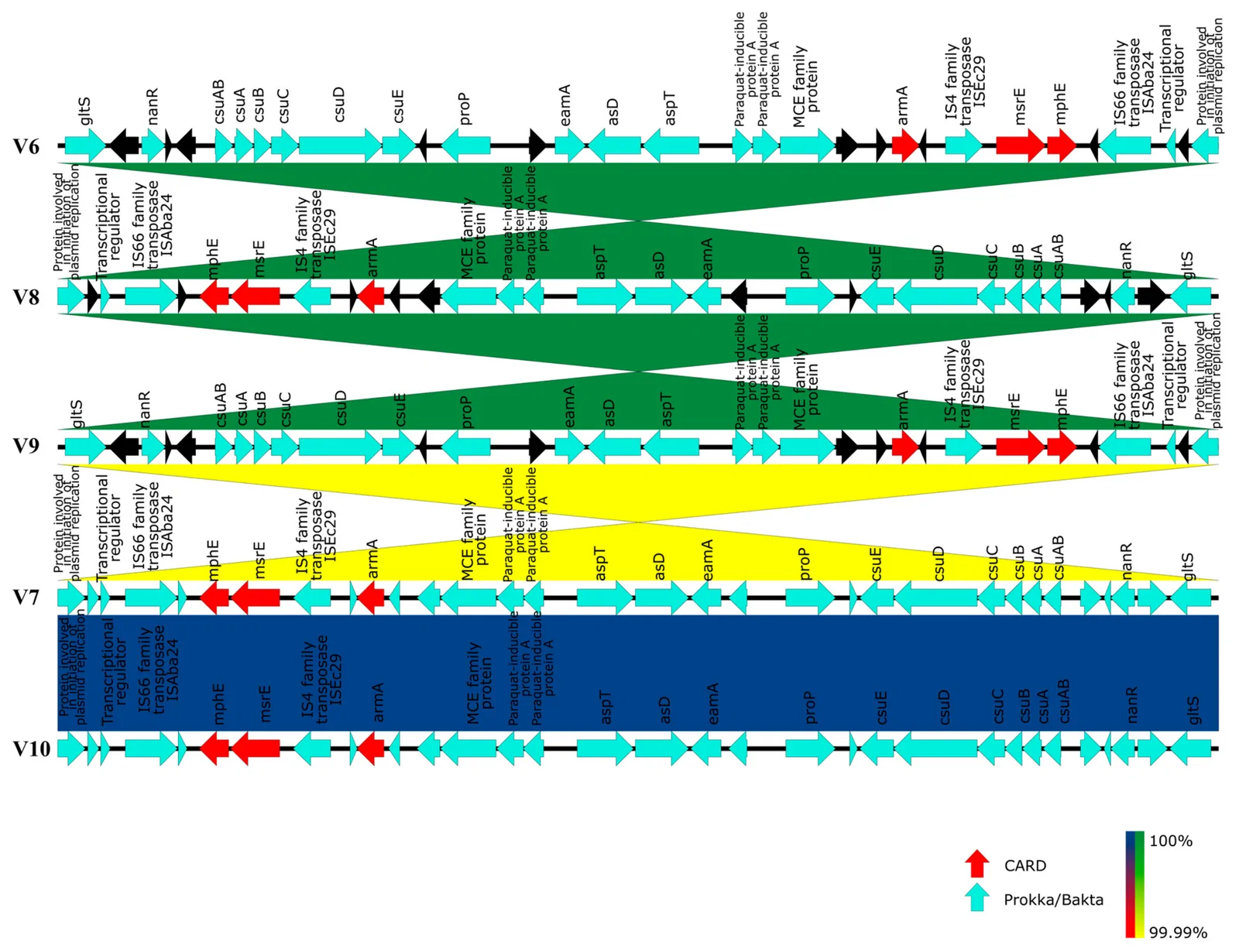
Comparative analysis of the genetic environment surrounding the *armA–msr(E)–mph(E)* multi-drug resistance region in CRAB isolates V6–V10. Genes are shown as arrows indicating transcriptional orientation. Resistance genes are highlighted in red, while other predicted coding sequences are highlighted in blue. Shaded regions represent pairwise BLASTn alignments, with colors indicating nucleotide sequence identity.

The genomic region surrounding the *armA–msr(E)–mph(E)* resistance cluster was highly conserved among all five isolates (Figure 5). The resistance genes were embedded within an approximately 35 kb multi-drug resistance region that contained the *csu* operon associated with biofilm formation, genes encoding membrane transporters and regulatory proteins, and multiple insertion sequence-associated genes. Overall nucleotide identity across the aligned region exceeded 99.99%, indicating an almost identical genetic organization in both ST2 and ST492 lineages. Minor differences were confined to the terminal parts of the contigs and were consistent with assembly fragmentation rather than genomic rearrangements.

AlienHunter predicted multiple putative horizontally acquired regions within the analyzed contigs (Figure S3). In all isolates, the *armA–msr(E)–mph(E)* resistance cluster was located within one of these compositionally atypical regions.

AcinetobacterPlasmidTyping identified a Rep3-T60 replication initiation protein on the same contig carrying the *armA–msr(E)–mph(E)* cluster in all isolates, whereas MOB-suite did not assign these contigs to a defined plasmid cluster (Table 1). ISFinder detected multiple conserved insertion sequences associated with this region, including ISAba24, ISEc29, and ISEc28/ISEc35.

Comparative analysis of the *bla*_OXA-72_-containing contigs demonstrated complete conservation of gene organization, with high nucleotide similarity across the aligned region between isolates V7 and V10 (Figure S4). The *bla*_OXA-72_ gene was located adjacent to genes encoding a DNA-binding protein, an MFS transporter, a Rep-3 domain-containing protein, a conserved domain protein, and a putative TonB-dependent receptor.

In contrast, the genetic environment surrounding *bla*_OXA-23_ could not be reconstructed because the corresponding contigs (~2.6 kb) were too short to enable reliable comparison of the flanking genomic regions.

Combined analyses using MOB-suite and AcinetobacterPlasmidTyping revealed different levels of evidence supporting plasmid association of the investigated resistance regions (Table 1), with a complete summary of all plasmid-associated contigs in Supplementary (Table S3).

The *bla*_OXA-72_-containing contigs of isolates V7 and V10 were assigned to the AB082 plasmid cluster by MOB-suite. AcinetobacterPlasmidTyping identified a Rep3-T1 replication initiation protein on the same contig (Table 1). BLASTn analysis showed that these contigs shared 100% query coverage and 99.99% nucleotide identity with several complete *A. baumannii* plasmids deposited in GenBank, including pAb64-p1 (CP195696.1), p2DB001 (CP087376.1), and unnamed2 (CP169793.1).

Conversely, although the *bla*_OXA-23_-containing contigs of the ST2 isolates were also assigned to the AB082 plasmid cluster, no plasmid replication initiation protein was detected on the corresponding *bla*_OXA-23_-containing contigs.

Along with the resistance-associated contigs, additional plasmid-associated contigs carrying Rep3-T1 (AC163 plasmid cluster) or RP-T1 replication initiation proteins were identified in several isolates, but antimicrobial resistance genes were not detected within these regions (Table S3).

ISFinder identified several insertion sequence elements associated with all investigated resistance regions (Table 1). The *bla*_OXA-23_ region was flanked by partial ISAba1 sequences at both contig ends, whereas the *bla*_OXA-72_ region contained IS66-family elements together with terminal ISAba31 fragments. IntegronFinder did not identify complete integrons associated with the investigated resistance loci.

## 3. Discussion

*A. baumannii* is a ubiquitous microorganism that has transitioned from a benign commensal into one of the most successful opportunistic pathogens, particularly within healthcare environments [3,13]. Over the past few decades, extensive research has focused on elucidating the mechanisms behind this evolutionary transition and developing strategies to combat this persistent pathogen. A comprehensive WGS analysis was conducted for the first time to elucidate recent trends in AMR and virulence among critically ill patients with *A. baumannii* LRTIs in ICU at the University Hospital of Split, Croatia.

Genomic analysis of Croatian CRAB isolates revealed the circulation of two MLST groups, ST2 and ST492, which exhibit highly conserved genomic backgrounds within each lineage but differ in carbapenemase content and the genetic organization of resistance-associated mobile elements. The cgMLST analysis clearly separated the study isolates into two phylogenetic lineages corresponding to Pasteur ST2 and ST492, fully supporting the MLST assignments. ST2 belongs to the internationally disseminated International Clone 2 (IC2), the predominant epidemic lineage responsible for healthcare-associated outbreaks worldwide and frequently associated with multi-drug- and carbapenem-resistant phenotypes [5,14,15]. Accordingly, the Croatian ST2 isolates clustered with publicly available genomes from several neighboring countries, indicating that they belong to the widespread regional IC2 population rather than representing a unique local lineage [12,14,15]. In contrast, the ST492 subclade exhibits the closest phylogenetic proximity to genomes originating from Serbia and Hungary. Although ST492 has been reported much less frequently than the globally disseminated ST2 lineage, it has previously been identified among Croatian hospital CRAB isolates [16,17]. In addition to Croatia, ST492 has also been reported among clinical isolates from Lebanon, Serbia, Romania, and Hungary [18,19,20,21], indicating that this lineage is geographically widespread but considerably less prevalent than IC2/ST2. The close phylogenetic relationship between the Croatian, Serbian, and Hungarian isolates observed in the present study further supports the regional circulation of ST492 in Southeast Europe.

The pairwise core-genome SNP analysis further strengthened the phylogenomic findings by quantitatively confirming the presence of two genetically distinct lineages corresponding to Pasteur ST2 and ST492. In addition, the observed SNP differences demonstrated that isolates sharing the same sequence type may still exhibit genomic diversity, highlighting the higher discriminatory power of whole-genome SNP analysis compared with MLST alone.

Despite belonging to two distinct sequence types, CRAB isolates exhibited highly similar resistome profiles, reflecting the accumulation of multiple acquired resistance determinants conferring resistance to all critical antimicrobial agents commonly reported among MDR *A. baumannii* lineages [22,23]. Both ST2 and ST492 isolates carried genes conferring resistance to aminoglycosides, macrolides, tetracyclines, sulfonamides, and β-lactams, together with several intrinsic and acquired multi-drug efflux systems, consistent with extensive adaptation to antimicrobial pressure. The conserved repertoire of multidrug efflux pumps identified in all isolates is in agreement with previous studies demonstrating that these transporters represent a core feature of the *A. baumannii* genome, although their clinical impact is largely determined by regulatory mutations leading to overexpression rather than gene presence alone [24]. The principal difference between the two lineages was the acquired carbapenemase gene. The globally disseminated ST2 isolates carried *bla*_OXA-23_, the worldwide predominant acquired carbapenemase and a characteristic of IC2, whereas the ST492 isolates harboured *bla*_OXA-72_, the only OXA-24/40-like carbapenemase variant reported in Croatia to date [16]. Although both enzymes confer carbapenem resistance, *bla*_OXA-23_ has become the dominant determinant globally, while *bla*_OXA-72_ has been increasingly associated with successful regional lineages circulating across Europe, Asia, and South America [25,26,27,28,29,30]. These findings further support the concept that similar carbapenem-resistant phenotypes may arise through the dissemination of distinct carbapenemase genes within different clonal backgrounds. In addition, all isolates carried the intrinsic *bla*_OXA-66_ gene together with multiple aminoglycoside resistance determinants, including the 16S rRNA methyltransferase *armA*, which confers high-level resistance to clinically important aminoglycosides [31,32]. While colistin resistance was observed in the present study, the molecular basis for this resistance was not identified. Colistin resistance is predominantly driven by alterations in Lipid A, the loss of specific porins, or the complete absence of lipopolysaccharide [13]. The chromosomal point mutations in the *pmrCAB* operon genes, which mediated colistin resistance in a previous study from Southeast Europe, were not detected in our research [14]. Notably, while sulbactam was previously considered intrinsically active against *A. baumannii*, the emergence of isolates requiring high antibiotic exposure in our study underscores the necessity of susceptibility testing for all clinical isolates [13]. These data raise serious concerns and highlight the immediate need to evaluate alternative therapeutic strategies, like antimicrobial peptides and phage therapy [31,32].

Comparative analyses of the genetic environments surrounding the investigated resistance determinants indicate that the multi-drug resistance region carrying *armA–msr(E)–mph(E)* was highly similar among all analyzed isolates, irrespective of sequence type. This finding is consistent with previous reports describing this multi-drug resistance region disseminated among epidemic MDR *A. baumannii* clones [33]. In contrast, the genetic context of the acquired carbapenemase genes differed between the two lineages. The combined results of different analyses consistently supported plasmid association of the *bla*_OXA-72_ region. The identification of a Rep3-T1 replication initiation protein, together with high sequence similarity to previously described *A. baumannii* plasmids, suggests that *bla*_OXA-72_ is carried by a conserved Rep3-family plasmid backbone. Rep3-family plasmids are well known as the vectors responsible for the dissemination of carbapenemases in *A. baumannii* [34,35,36]. By contrast, the genomic location of *bla*_OXA-23_ could not be resolved unambiguously because the corresponding contigs were too short for complete structural reconstruction. Nevertheless, the detection of terminal ISAba1 fragments supports previous observations that *bla*_OXA-23_ is commonly associated with ISAba1-containing transposon structures, which facilitate both gene expression and horizontal dissemination [13]. Consequently, the complete genetic environment of *bla*_OXA-23_ could not be reconstructed using short-read sequencing alone, and its genomic localization should therefore be interpreted with caution.

Furthermore, profiling of virulence determinants detailed the pathogenic potential of *A. baumannii*. A large array of virulence genes, including genes encoding different steps in pathogenesis (adherence (*fim*, *pil*), invasion (*plc*, *plcD)*, host–pathogen interaction (*lpx, lps*), persistence (*gal*, *pgi*, *pse*, *tvi*), and, notably, biofilm formation (*aba*, *adeFGH*, *bap*, *csu*)), was likewise highly conserved and largely reflected the phylogenetic relationships of the isolates [1,7,34]. Additionally, genotypic validation of the *bau*, *bas*, *ent*, and *bar* gene clusters confirmed active acinetobactin production, a major siderophore that plays a critical role in the LRT colonization and infection [1].

Such gene conservation is expected because many of these determinants represent core virulence factors required for environmental persistence, colonization of abiotic surfaces, and successful establishment of hospital-associated infections [7,27,28,34].

The similarity of virulence profiles between ST2 and ST492 further suggests that the differences observed between these lineages are primarily associated with antimicrobial resistance rather than with major variations in virulence potential. The concurrent presence of extensive AMR genes and a robust virulence profile observed in this research aligns with data reported for other bacterial species. Furthermore, the genetic architecture driving these traits strongly suggests a functional or structural linkage between resistance and virulence mechanisms [37]. Overall, these findings provide a comprehensive genomic overview of Croatian CRAB isolates and improve our understanding of the population structure, antimicrobial resistance, and virulence characteristics of the two predominant lineages circulating in this setting.

However, several limitations of this study should nevertheless be acknowledged. Genome assemblies were generated exclusively from short-read Illumina sequencing, preventing complete reconstruction of plasmids and transposable elements and limiting precise localization of certain resistance determinants, particularly *bla*_OXA-23_. Consequently, the complete genetic environment of this carbapenemase could not be resolved. Future studies integrating long-read sequencing with transcriptomic and functional analyses will enable the complete reconstruction of mobile resistance platforms and facilitate the elucidation of unresolved resistance mechanisms, including the molecular basis of colistin resistance observed in isolate V10. Additionally, due to its cross-sectional design, we could only evaluate strains isolated within a specific timeframe. However, our phenotypic analysis included all isolates from a single setting, and a significant proportion of these were analyzed using WGS, providing an accurate representation of the strains present among selected patients in the hospital’s ICU. Nevertheless, further research integrating patients’ clinical and therapeutic data is warranted to provide a deeper insight into the relationship between the observed virulence, antimicrobial resistance, and clinical outcomes.

## 4. Materials and Methods

### 4.1. Study Design and Inclusion Criteria

Over a 1-year period (January 2023 to January 2024), 79 patients in the ICU met the diagnostic criteria for LRTI, specifically VAP or VAT in ICU.

Diagnosis of LRTIs was based on clinical, microbiological, and radiological criteria [38,39,40]. Following the clinical identification of suggestive LRTI symptoms, acute pulmonary changes were radiologically investigated. The main distinction between VAT and VAP is that VAT does not fulfill the radiological criteria for LRTI.

To meet the microbiological criteria, *A. baumannii* had to be isolated from bronchial specimens (with a significant threshold of ≥10^4^ colony-forming units [CFU]/mL) or from tracheal aspirates (with a significant threshold of ≥10^6^ CFU/mL) to confirm the diagnosis.

### 4.2. Cultivation, Bacterial Identification, and Antimicrobial Susceptibility Testing

Bronchial and tracheal aspirates were quantitatively inoculated onto blood agar (BA) and nonselective CHROMagar Orientation (CHROMagar, Paris, France) medium. Following incubation under capnophilic conditions (10% CO_2_) at 37 °C for 24 h, plates were examined for bacterial growth. If colonies were visible, standard microbiological identification was conducted using matrix-assisted laser desorption ionization mass spectrometry (MALDI-TOF MS, MALDI Biotyper^®^ sirius, Bruker, Bremen, Germany). Plates without growth were reincubated for an additional 24 h under the same conditions.

Antimicrobial susceptibility testing (AST) was performed using the Kirby Bauer disk diffusion method (Liofilchem, Roseto degli Abruzzi, Italy). The panel included quinolones (CIP, and LEV), carbapenems (IMI, and MEM), SXT, and aminoglycosides (AMK, GEN, and TOB). Results were interpreted as susceptible (S), susceptible with increased exposure (I), or resistant (R) according to the European Committee on Antimicrobial Susceptibility Testing guidelines (EUCAST, version 13.0) [41].

AST to colistin was performed using the reference broth dilution (BD) method and interpreted according to EUCAST guidelines [42].

Additionally, AST for SAM was performed and interpreted according to the Clinical and Laboratory Standards Institute (CLSI) guidelines, as there are no breakpoints provided by EUCAST [43].

*A. baumannii* ATCC 19606 was used as the standard quality control strain.

The multi-drug resistance (MDR), extensively drug resistance (XDR), and pan-drug resistance (PDR) were defined according to Magiorakos et al. [23].

### 4.3. Genomic DNA Extraction and Whole-Genome Sequencing

Five representative isolates (5/18; 27.8%) were selected to capture phenotypic diversity (including the only colistin-resistant isolate) and the CRAB clones which circulated during the study period. Sixteen isolates shared an identical phenotypic resistance pattern, from which three isolates from different time periods were randomly selected for WGS. An additional isolate was chosen for WGS because it was resistant to colistin, while the final isolate was selected based on an increased-exposure susceptible profile to ampicillin/sulbactam (SAM). Genomic DNA (isolates V6–V10) was extracted using the NucleoSpin Microbial DNA Kit (Macherey-Nagel, Cambridge, UK). The concentration and quality of the extracted DNA were assessed using a NanoDrop^®^ 1000 Spectrophotometer (Thermo Scientific, Waltham, MA, USA). Genomic DNA was sent to Novogene (Cambridge, UK) for WGS. DNA libraries were prepared using the NEBNext^®^ DNA Library Prep Kit (Illumina, San Diego, CA, USA) according to the manufacturer’s instructions. After library quantification by Qubit fluorometer and qPCR, paired-end sequencing (2 × 150 bp) was performed on the Illumina NovaSeq 6000 platform. Raw reads were subjected to quality control to remove adapter contamination, with reads containing more than 10% ambiguous nucleotides (N) and reads with more than 50% of bases having a Phred score ≤ 5.

### 4.4. Genome Analysis

Raw Illumina paired-end reads were assembled de novo using SPAdes genome assembler (v4.0.0) in isolate mode. The resulting contigs were annotated with Prokka (v1.14.6). Functional genomic characterization was performed using the ABRicate tool (v1.0.1). Assembled genomes were screened against ABRicate databases, including CARD, ARG-ANNOT, Resfinder, and VFDB, to identify antimicrobial resistance genes (ARGs), virulence factors, and other relevant genetic elements [44,45,46]. To complement the resistance gene analysis, assembled genomes were additionally screened using AMRFinderPlus v4.2.7 (NCBI) with the reference database version 2026-05-15.1 to identify acquired ARGs and chromosomal point mutations associated with antimicrobial resistance [47]. To investigate the genetic basis of COL resistance, the COL-resistant isolate V10 was compared with the closely related COL-susceptible isolate V7 using WGS data. Comparative genomic analysis was performed using Snippy to identify nucleotide variations between the two isolates. Known COL resistance-associated determinants, including the *pmrAB*, *phoPQ*, *pmrC/eptA*-like, *lpxA*, *lpxC*, *lpxD,* and *mcr* genes, were examined using genome annotations generated by Prokka together with ABRicate and AMRFinderPlus. The presence of insertion sequence elements potentially associated with COL resistance was additionally assessed using ISFinder. Furthermore, V10 was compared with publicly available genomes exhibiting COL-resistant (GCA_009759985.1; MIC = 8 mg/L) and COL-susceptible (GCA_009760525.1; MIC = 0.5 mg/L) phenotypes to identify genomic features potentially associated with the resistant phenotype.

To assess genomic relatedness between the five *A. baumannii* isolates and 1012 publicly available complete *A. baumannii* RefSeq genomes, and to place the Croatian isolates within the broader genomic diversity of the species, Mash (v2.3) was used, followed by Average Nucleotide Identity (ANI) analysis of the closest genomes using fastANI (v1.34). Dendrograms were constructed using the 10 closest RefSeq genomes identified for each isolate. To further quantify the genomic relatedness among the five sequenced isolates, pairwise core-genome single-nucleotide polymorphism (SNP) analysis was performed using Snippy v4.6.0. Raw Illumina reads from isolates V6–V10 were mapped against the complete chromosome of *A. baumannii* ACICU (NC_010611.1). A common core-genome alignment was generated using snippy-core, and pairwise SNP distances were calculated using snp-dists v1.2.0. The resulting SNP distance matrix was visualized as a heatmap in R (v4.5.1).

To determine the clonal background of the sequenced isolates, sequence types were assigned using both the Pasteur and Oxford *A. baumannii* MLST schemes [45]. In isolates containing multiple *gdhB* alleles, alternative Oxford sequence types were also reported.

To infer phylogenetic relationships among the five sequenced isolates based on their shared core genome, pangenome analysis was performed using Panaroo (v1.2.10). A core genome alignment was generated from the assembled genomes and used for phylogenetic reconstruction. Maximum-likelihood phylogenetic analysis was conducted in IQ-TREE v2.3.6 with automatic model selection implemented in ModelFinder. The best-fitting nucleotide substitution model according to the Bayesian Information Criterion (BIC) was TIM2 + F + I. Branch support was assessed using 1000 ultra-fast bootstrap replicates. The resulting phylogenetic tree was visualized and annotated with sequence type (ST) profiles in iTOL (v7.2.2) together with antimicrobial resistance and virulence gene presence/absence data [48].

To investigate the regional phylogenetic relationships of the study isolates, five Croatian genomes were compared with 294 publicly available *A. baumannii* genomes retrieved from the PubMLST database (accessed on 22 June 2026). The dataset comprised isolates originating from Croatia and neighboring countries, including Bosnia and Herzegovina, Serbia, Montenegro, Slovenia, Slovakia, Italy, and Hungary. All publicly available genomes meeting these geographical criteria at the time of data retrieval were included to provide the broadest possible regional phylogenetic context for the Croatian isolates. Core genome MLST (cgMLST) analysis was performed using the PubMLST platform, and phylogenetic trees were generated using the FastTree algorithm. Metadata, including country of origin, isolation source, and Oxford and Pasteur sequence types, were exported and visualized in iTOL (v7.2.2). Final figures were prepared in Inkscape (v1.3.2).

To investigate the genetic context of the major antimicrobial resistance determinants, genetic environments surrounding the carbapenemase genes *bla*_OXA-23_ and *bla*_OXA-72_, as well as the aminoglycoside and macrolide resistance genes *armA*, *msr(E)*, and *mph(E)*, were investigated through comparative genomic analyses. Contigs carrying the resistance loci were extracted from genome assemblies using SeqKit (v2.1.0). For visualization purposes, homologous regions encompassing the resistance genes and adjacent insertion sequence elements were trimmed to comparable lengths using SeqKit subseq prior to Easyfig analysis. The extracted regions were re-annotated using Prokka and compared using Easyfig [49]. BLASTn alignments were generated between genomic regions and visualized as shaded blocks according to nucleotide sequence identity. Gene annotations obtained from Prokka, Bakta, CARD, and ISFinder were incorporated into the final comparative figures, finalized and formatted in Inkscape v1.3.2.

To identify putative horizontally acquired genomic regions associated with major resistance loci, AlienHunter (v1.7.8) was used. Analyses were performed for contigs carrying the *armA–msr(E)–mph(E)* multi-drug resistance region. Although the *bla*_OXA-23_- and *bla*_OXA-72_-containing contigs were also screened, only the *bla*_OXA-72_ region was suitable for comparative genomic visualization because the *bla*_OXA-23_ contigs were too short to reconstruct their complete genetic environment.

To investigate the potential mobility of resistance determinants, insertion sequences, integrons, and plasmid-associated contigs were characterized using ISFinder, IntegronFinder, MOB-suite, and AcinetobacterPlasmidTyping. The putative plasmid-derived contig was compared against the NCBI database using BLASTn (megablast) to determine its similarity to known *A. baumannii* plasmid sequences.

## 5. Conclusions

This study provides a comprehensive whole-genome characterization of representative CRAB isolates circulating in a Croatian ICU and demonstrates the co-circulation of two high-risk lineages, Pasteur ST2 and ST492. By integrating complementary genomic analyses, including phylogenomic reconstruction, pairwise core-genome SNP analysis, resistome, virulome, and genetic context characterization, the study provides new insights into the genomic diversity of circulating CRAB lineages, the organization of major antimicrobial resistance determinants, and their regional phylogenetic relatedness. The findings highlight the value of WGS-based genomic surveillance for monitoring the emergence and regional dissemination of high-risk CRAB lineages and support its application in infection prevention and antimicrobial resistance surveillance.

## Figures and Tables

**Table 1:** Plasmid-associated features and insertion sequence elements associated with antimicrobial resistance genes in CRAB isolates V6–V10.

Antimicrobial Resistance Genes	Isolate(s)	Contig(s)	MOB-Suite Plasmid Cluster	Rep Type Detected on the Same Contig (Acinetobacter PlasmidTyping)	Insertion Sequence(s)
*armA–msr(E)–mph(E)*	V6, V8, V9	NODE22/NODE12	Not assigned	Rep3-T60	ISAba24, ISEc29, ISEc28, IS15/IS26-like
	V7, V10	NODE30	Not assigned	Rep3-T60	ISAba24, ISEc29, ISEc28, IS15/IS26-like, terminal ISAba1
*bla*_OXA-23_	V6	NODE56	AB082	Not detected	Partial ISAba1 at both contig ends
	V8	NODE51	AB082	Not detected	Partial ISAba1 at both contig ends
	V9	NODE57	AB082	Not detected	Partial ISAba1 at both contig ends
*bla*_OXA-72_	V7	NODE49	AB082	Rep3-T1	IS66-family elements and terminal ISAba31
	V10	NODE50	AB082	Rep3-T1	IS66-family elements and terminal ISAba31

## Data Availability

The data from this study have been deposited in European Nucleotide Archive (ENA) under project PRJEB102320, and the accession numbers for each isolate are as follows: ERS27185635, ERS27185636, ERS27185637, ERS27185638, and ERS27185639.

## Supplementary Materials

The following supporting information can be downloaded at: https://www.mdpi.com/article/10.3390/antibiotics15080814/s1, Figure S1: Comparative genomic clustering of the five CRAB isolates. (A) MASH-based clustering. (B) fastANI-based clustering; Figure S2: Pairwise core-genome SNP distance heatmap among the five sequenced CRAB isolates; Figure S3: AlienHunter MDR; Figure S4: Comparative analysis of blaOXA-72–containing contigs; Table S1: Metadata of the whole genome sequenced CRAB isolates V6–V10 from Croatia; Table S2: Pairwise core-genome SNP distances among CRAB isolates V6–V10; Table S3: Summary of plasmid-associated features identified by MOB-suite, AcinetobacterPlasmidTyping and ISFinder in CRAB isolates V6–V10.

## Abbreviations

The following abbreviations are used in this manuscript:

ICU	Intensive Care Unit
WGS	Whole-genome sequencing
CRAB	Carbapenem- resistant *Acinetobacter baumannii*
MLST	Multi-locus sequence typing
XDR	Exstensively drug-resistant
PDR	Pandrug- resistant
HAI	Healthcare-associated infection
WHO	World Health Organisation
OXA	Oxacillinase
LRTI	Lower respiratory tract infection
IC2	International Clone 2
EUCAST	European Committee on Antimicrobial Susceptibility Testing
CLSI	Clinical and Laboratory Standards Institute
NCBI	National Center for Biotechnology Information
ST	Sequence Type
IS	Insertion sequence

## References

[1] Yao Y., Chen Q., Zhou H. (2023). Virulence factors and pathogenicity mechanisms of *Acinetobacter baumannii* in respiratory infectious diseases. Antibiotics.

[2] World Health Organization (2017). Prioritization of Pathogens to Guide Discovery, Research and Development of New Antibiotics for Drug-Resistant Bacterial Infections, Including Tuberculosis.

[3] Sarshar M., Behzadi P., Scribano D., Palamara A.T., Ambrosi C. (2021). *Acinetobacter baumannii*: An ancient commensal with weapons of a pathogen. Pathogens.

[4] Lucidi M., Visaggio D., Migliaccio A., Capecchi G., Visca P., Imperi F., Zarrilli R. (2024). Pathogenicity and virulence of *Acinetobacter baumannii*: Factors contributing to fitness in healthcare settings and the infected host. Virulence.

[5] Goic-Barisic I., Kovacic A., Medic D., Jakovac S., Petrovic T., Tonkic M., Novak A., Rubic Z., Radic M., Milosavljević B. (2021). Endemicity of OXA-23 and OXA-72 in clinical isolates of *Acinetobacter baumannii* from three neighbouring countries in Southeast Europe. J. Appl. Genet..

[6] Nguyen M., Joshi S.G. (2021). Carbapenem resistance in *Acinetobacter baumannii* and its importance in hospital-acquired infections: A scientific review. J. Appl. Microbiol..

[7] Wu C., Yuan Y., Tang S., Liu C., He C. (2024). Clinical and microbiological features of a cohort of patients with *Acinetobacter baumannii* bloodstream infections. Eur. J. Clin. Microbiol. Infect. Dis..

[8] Sutimbekova N., Bissenova N., Dusmagambetov M., Saltabayeva U., Utegenova A., Smanova G., Igissenova A., Rakhimzhanova F., Askarova N., Duissebekova G. (2026). Phenotype-genotype discordance in antimicrobial resistance of *Acinetobacter baumannii*: Implications for diagnostics and surveillance. Pathogens.

[9] Tiku V. (2022). *Acinetobacter baumannii*: Virulence strategies and host defense mechanisms. DNA Cell Biol..

[10] Kazemi Najafabadi M., Soltani R. (2024). Carbapenem-resistant *Acinetobacter baumannii* and ventilator-associated pneumonia: Epidemiology, risk factors, and current therapeutic approaches. J. Res. Pharm. Pract..

[11] Zhang Y.Y., Liang Z.X., Li C.S., Chang Y., Ma X.Q., Yu L., Chen L.-A. (2018). Whole-genome analysis of an extensively drug-resistant *Acinetobacter baumannii* strain XDR-BJ83: Insights into the mechanisms of resistance of an ST368 strain from a tertiary care hospital in China. Microb. Drug Resist..

[12] Goic-Barisic I., Towner K.J., Kovacic A., Sisko-Kraljevic K., Tonkic M., Novak A., Punda-Polic V. (2011). Outbreak in Croatia caused by a new carbapenem-resistant clone of *Acinetobacter baumannii* producing OXA-72 carbapenemase. J. Hosp. Infect..

[13] Roca I., Espinal P., Vila-Farrés X., Vila J. (2012). The *Acinetobacter baumannii* oxymoron: Commensal hospital dweller turned pan-drug-resistant menace. Front. Microbiol..

[14] Goic-Barisic I., Music M.S., Drcelic M., Tuncbilek S., Akca G., Jakovac S., Tonkić M., Hrenovic J. (2023). Molecular characterisation of colistin- and carbapenem-resistant clinical isolates of *Acinetobacter baumannii* from Southeast Europe. J. Glob. Antimicrob. Resist..

[15] Castanheira M., Mendes R.E., Gales A.C. (2023). Global epidemiology and mechanisms of resistance of *Acinetobacter baumannii-calcoaceticus* complex. Clin. Infect. Dis..

[16] Lukić-Grlić A., Kos M., Žižek M., Luxner J., Grisold A., Zarfel G., Bedenić B. (2019). Emergence of carbapenem-hydrolyzing oxacillinases in *Acinetobacter baumannii* in children from Croatia. Chemotherapy.

[17] Bedenić B., Nađ M., Bratić V., Pavlović D.B., Kasalo M., Dobrić M., Pino R.A.d., Burgwinkel T., Grisold A., Luxner J. (2026). Carbapenem-resistant *Acinetobacter baumannii* in Zagreb, Croatia, in the post-COVID-19 pandemic period: Resistance trends and mechanisms. Microorganisms.

[18] Al Bayssari C., Dabboussi F., Hamze M., Rolain J.-M. (2015). Emergence of carbapenemase-producing *Pseudomonas aeruginosa* and *Acinetobacter baumannii* in livestock animals in Lebanon. J. Antimicrob. Chemother..

[19] Luković B., Gajić I., Dimkić I., Kekić D., Zornić S., Pozder T., Radisavljevic S., Opavski N., Kojić M., Ranin L. (2020). The first nationwide, multicenter study of *Acinetobacter baumannii* recovered in Serbia: Emergence of OXA-72, OXA-23 and NDM-1-producing isolates. Antimicrob. Resist. Infect. Control.

[20] Gheorghe I., Barbu I.C., Surleac M., Sârbu I., Popa L.I., Paraschiv S., Feng Y., Lazăr V., Chifiriuc M.C., Oţelea D. (2021). Subtypes, resistance and virulence platforms in extended-drug-resistant *Acinetobacter baumannii* Romanian isolates. Sci. Rep..

[21] Balázs B., Tóth Z., Nagy J.B., Majoros L., Tóth Á., Kardos G. (2022). Faecal carriage of carbapenem-resistant *Acinetobacter baumannii*: Comparison to clinical isolates from the same period (2017–2019). Pathogens.

[22] Perez F., Hujer A.M., Hujer K.M., Decker B.K., Rather P.N., Bonomo R.A. (2007). Global challenge of multidrug-resistant *Acinetobacter baumannii*. Antimicrob. Agents Chemother..

[23] Magiorakos A.-P., Srinivasan A., Carey R.B., Carmeli Y., Falagas M.E., Giske C.G., Harbarth S., Hindler J.F., Kahlmeter G., Olsson-Liljequist B. (2012). Multidrug-resistant, extensively drug-resistant and pandrug-resistant bacteria: An international expert proposal for interim standard definitions for acquired resistance. Clin. Microbiol. Infect..

[24] Coyne S., Courvalin P., Périchon B. (2011). Efflux-mediated antibiotic resistance in *Acinetobacter* spp.. Antimicrob. Agents Chemother..

[25] Jeon W.J., Kim Y.J., Seo J.H., Yoo J.S., Moon D.C. (2024). Genomic analysis of carbapenem-resistant *Acinetobacter baumannii* isolated from bloodstream infections in South Korea. Antibiotics.

[26] Povilonis J., Seputiene V., Krasauskas R., Juskaite R., Miskinyte M., Suziedelis K., Suziedeliene E. (2013). Spread of carbapenem-resistant *Acinetobacter baumannii* carrying a plasmid with two genes encoding OXA-72 carbapenemase in Lithuanian hospitals. J. Antimicrob. Chemother..

[27] Kuo S.C., Yang S.P., Lee Y.T., Chuang H.C., Chen C.P., Chang C.L., Chen T.L., Lu P.L., Hsueh P.R., Fung C.P. (2013). Dissemination of imipenem-resistant *Acinetobacter baumannii* with new plasmid-borne *bla*_OXA-72_ in Taiwan. BMC Infect. Dis..

[28] Barnaud G., Zihoune N., Ricard J.D., Hippeaux M.C., Eveillard M., Dreyfuss D., Branger C. (2010). Two sequential outbreaks caused by multidrug-resistant *Acinetobacter baumannii* isolates producing OXA-58 or OXA-72 oxacillinase in an intensive care unit in France. J. Hosp. Infect..

[29] Saavedra S.Y., Cayo R., Gales A.C., Leal A.L., Saavedra C.H. (2014). Early dissemination of OXA-72-producing *Acinetobacter baumannii* strain in Colombia: A case report. Braz. J. Infect. Dis..

[30] Vasconcelos A.T., Barth A.L., Zavascki A.P., Gales A.C., Levin A.S., Lucarevschi B.R., Cabral B.G., Brasiliense D.M., Rossi F., Furtado G.H. (2015). The changing epidemiology of *Acinetobacter* spp. producing OXA carbapenemases causing bloodstream infections in Brazil: A BrasNet report. Diagn. Microbiol. Infect. Dis..

[31] Lopes B.S., Hanafiah A., Nachimuthu R., Muthupandian S., Md Nesran Z.N., Patil S. (2022). The role of antimicrobial peptides as antimicrobial and antibiofilm agents in tackling the silent pandemic of antimicrobial resistance. Molecules.

[32] Sukri A., Lopes B.S., Hanafiah A. (2026). Phage therapy in combating multidrug-resistant Gram-negative pathogens: A scoping review. Pharmaceuticals.

[33] Huang X., Ning N., Li D., Chen S., Zhang L., Wang H., Bao C., Yang X., Li B., Wang H. (2024). Molecular epidemiology of *Acinetobacter baumannii* during COVID-19 at a hospital in northern China. Ann. Clin. Microbiol. Antimicrob..

[34] Unlu Celebi S., Yalcin S., Kurt Azap O., Uskudar Guclu A. (2026). Genomic insights into the genetic diversity, resistance determinants, and plasmid content of carbapenem-resistant *Acinetobacter baumannii* clinical isolates. Arch. Microbiol..

[35] Liu M., Yan C., Li L., Huang L. (2026). Analysis of clinical characteristics and whole-genome sequencing of carbapenem-resistant *Acinetobacter baumannii* isolates from pediatric patients in Guangxi, China. BMC Microbiol..

[36] Lam M.M.C., Hamidian M. (2024). Examining the role of *Acinetobacter baumannii* plasmid types in disseminating antimicrobial resistance. npj Antimicrob. Resist..

[37] Zhang L., Levy K., Trueba G., Cevallos W., Trostle J., Foxman B., Marrs C.F., Eisenberg J.N.S. (2015). Effects of selection pressure and genetic association on the relationship between antibiotic resistance and virulence in *Escherichia coli*. Antimicrob. Agents Chemother..

[38] Martin-Loeches I., Reyes L.F., Nseir S., Ranzani O., Povoa P., Diaz E., Schultz M.J., Rodríguez A.H., Serrano-Mayorga C.C., De Pascale G. (2023). European Network for ICU Related Respiratory Infections (ENIRRIs): A multinational, prospective, cohort study of nosocomial lower respiratory tract infections. Intensive Care Med..

[39] Ramírez Estrada S., Lagunes L., Peña López Y., Vahedian Azimi A., Nseir S., Arvaniti K., Bastug A., Totorika I., Oztoprak N., Bouadma L. (2018). Assessing predictive accuracy for outcomes of ventilator-associated events in an international cohort: The EUVAE study. Intensive Care Med..

[40] Foucrier A., Dessalle T., Tuffet S., Federici L., Dahyot-Fizelier C., Barbier F., Pottecher J., Monsel A., Hissem T., Lefrant J.-Y. (2023). Association between combination antibiotic therapy as opposed to monotherapy and outcomes of ICU patients with *Pseudomonas aeruginosa* ventilator-associated pneumonia: An ancillary study of the iDIAPASON trial. Crit. Care.

[41] EUCAST Clinical Breakpoints and Dosing of Antibiotics. https://www.eucast.org/clinical_breakpoints.

[42] Matuschek E., Åhman J., Webster C., Kahlmeter G. (2018). Antimicrobial susceptibility testing of colistin—Evaluation of seven commercial MIC products against standard broth microdilution for *Escherichia coli*, *Klebsiella pneumoniae*, *Pseudomonas aeruginosa*, and *Acinetobacter* spp.. Clin. Microbiol. Infect..

[43] Clinical and Laboratory Standards Institute (2018). Performance Standards for Antimicrobial Susceptibility Testing.

[44] Feldgarden M., Brover V., Haft D.H., Prasad A.B., Slotta D.J., Tolstoy I., Tyson G.H., Zhao S., Hsu C.-H., McDermott P.F. (2019). Validating the AMRFinder tool and resistance gene database using antimicrobial resistance genotype–phenotype correlations. Antimicrob. Agents Chemother..

[45] Chen L., Zheng D., Liu B., Yang J., Jin Q. (2016). VFDB 2016: Hierarchical and refined dataset for big data analysis—10 years on. Nucleic Acids Res..

[46] Jia B., Raphenya A.R., Alcock B., Waglechner N., Guo P., Tsang K.K., Lago B.A., Dave B.M., Pereira S., Sharma A.N. (2017). CARD 2017: Expansion and model-centric curation of the Comprehensive Antibiotic Resistance Database. Nucleic Acids Res..

[47] Feldgarden M., Brover V., Gonzalez-Escalona N., Frye J.G., Haendiges J., Haft D.H., Hoffmann M., Pettengill J.B., Prasad A.B., Tillman G.E. (2021). AMRFinderPlus and the Reference Gene Catalog facilitate examination of the genomic links among antimicrobial resistance, stress response, and virulence. Sci. Rep..

[48] Letunic I., Bork P. (2021). Interactive Tree of Life (iTOL) v5: An online tool for phylogenetic tree display and annotation. Nucleic Acids Res..

[49] Sullivan M.J., Petty N.K., Beatson S.A. (2011). Easyfig: A genome comparison visualizer. Bioinformatics.

